# Behavioral Tagging: A Translation of the Synaptic Tagging and Capture Hypothesis

**DOI:** 10.1155/2015/650780

**Published:** 2015-08-25

**Authors:** Diego Moncada, Fabricio Ballarini, Haydée Viola

**Affiliations:** ^1^Instituto de Biologia Celular y Neurociencias “Dr. Eduardo De Robertis”, Facultad de Medicina, Universidad de Buenos Aires, C1121ABG Buenos Aires, Argentina; ^2^Departamento de Fisiologia, Biologia Molecular y Celular Dr. Hector Maldonado, Facultad de Ciencias Exactas y Naturales, Universidad de Buenos Aires, C1428EGA Buenos Aires, Argentina

## Abstract

Similar molecular machinery is activated in neurons following an electrical stimulus that induces synaptic changes and after learning sessions that trigger memory formation. Then, to achieve perdurability of these processes protein synthesis is required for the reinforcement of the changes induced in the network. The synaptic tagging and capture theory provided a strong framework to explain synaptic specificity and persistence of electrophysiological induced plastic changes. Ten years later, the behavioral tagging hypothesis (BT) made use of the same argument, applying it to learning and memory models. The hypothesis postulates that the formation of lasting memories relies on at least two processes: the setting of a learning tag and the synthesis of plasticity related proteins, which once captured at tagged sites allow memory consolidation. BT explains how weak events, only capable of inducing transient forms of memories, can result in lasting memories when occurring close in time with other behaviorally relevant experiences that provide proteins. In this review, we detail the findings supporting the existence of BT process in rodents, leading to the consolidation, persistence, and interference of a memory. We focus on the molecular machinery taking place in these processes and describe the experimental data supporting the BT in humans.

## 1. Introduction

Animals have the ability to modify their behavior by learning and also the ability to retain the learned information over long periods of time in their memory [1]. This cognitive function is responsible for remembering events, facts, situations, places, objects, and motor skills [2]. All this information leads the individuals to behave according to the circumstances by adapting to the uncertain conditions of the environment.

Memory formation process displays some main features: it enables the retention of specific information about the world, it goes initially by a fragile state being the information slowly consolidated into a long-term memory, and it can eventually persist for long-lasting period of time even through all animal's life [3–6]. The resemblance of these general attributes of memory to those observed in synaptic long-term potentiation (LTP) and long-term depression (LTD) models of plasticity [7–10] leads to the postulation of the synaptic plasticity and memory hypothesis [11, 12]. It states that an activity-dependent plastic modification is induced at appropriate synapses during memory formation. Thus, plastic changes must occur in those brain areas where memory is being processed and are both necessary and sufficient for the storage of that information [11].

It is now widely accepted that neural activity induced by learning triggers changes in the strength of synaptic connections within the brain. In that sense, several experimental reports based on diverse associative, spatial, recognition, or motor memory paradigms support this statement [13–20]. Although memory is a complex property of the entire organism, multiple attempts have been made to correlate memory with electrophysiological models of synaptic plasticity [11]. In this review, we focus on the fact that they exhibit short-term phases and that they require protein synthesis for memory consolidation and synaptic plasticity maintenance in order to establish their respective long-term phases [21, 22]. However, how can the neuronal machinery assure the delivery of these proteins to specific sites where plasticity should be held? Using models of synaptic plasticity, Frey and Morris [23] postulated the “synaptic tagging and capture” hypothesis (STC), which declares that LTP involves the local tagging of synapses at the moment of its induction. Then, those tags can capture plasticity-related proteins (PRPs) synthesized in the soma allowing the stabilization of the potentiation for long periods of time. The hypothesis was tested initially using hippocampal slice preparations and it was recently demonstrated in the living rat [23, 24].

Lasting changes in synaptic plasticity strength and also in memory storage persistence are not only dependent on the characteristics of the stimuli that induce these changes. Events happening before or after these stimuli can also exert influence on synaptic plasticity and memory storage. This late-associative phenomenon was first seen by registering the change on a postsynaptic response triggered by stimulation. This is due to the action of a second spatial and temporally distant stimulation to another neuronal pathway targeting a common population of cells. A typical STC protocol shows that a stimulation that normally leads to early-term potentiation (e-LTP) can also induce a late-phase LTP (L-LTP) if a separate convergent pathway is strongly tetanized within a specific time-window [23, 25, 26]. Similar results were also observed applying low frequency stimulations that induced LTD [27–29]. In all those works the effect was abolished by the application of anisomycin, suggesting that the process is protein synthesis-dependent. In sum, STC postulated that strong stimuli synthesized PRPs could be used by independent tags if they converge in a given place and at a certain time. Even more, in a revisited version of the hypothesis, local protein synthesis and compartmentalization within a neuron are important factors for the setting of clustered plasticity [30, 31].

Late-associative effects induced by two different stimuli, first described in synaptic plasticity assays, were then translated into learning paradigms and opened a new approach to think about the process of LTM formation. It has been shown that short-lasting memory (STM), induced by a weak training, can be consolidated into a long-term memory (LTM) if animals experience a strong event in a critical time-window around the weak training. This process that depends on protein synthesis induced by the strong associated experience was originally named “behavioral tagging” (BT) [32]. In analogy to STC postulates, BT suggested that the weak training sets a learning tag where the PRPs provided by the strong event would be captured in order to establish a persistent mnemonic trace. Therefore, signaling “where” to store the information seems as important as the synthesis of PRPs, in order to allow the formation of a lasting memory.

In this review we explain why BT is a suitable model to explain LTM formation. We detail the postulates of tagging and capture hypothesis and we describe the action of a novel experience over the formation of several independent LTMs, making emphasis on the mechanism involved in this process. In addition, we highlight the implications of BT process in protocols of interference where a different learning competes to consolidate their own memory. And finally, we describe experiments suggesting the involvement of BT process in the persistence of consolidated memories as well as in the formation of human's LTM.

## 2. Postulates of Tagging and Capture Hypothesis

The principal idea underlying the process of tagging and capture is that PRPs are used to yield long-lasting changes when they are captured by specific tags. This mechanism was revealed dissecting the step of tag setting from the step of PRPs synthesis. Protocols using a weak stimulus given close to a strong one helped to unveil the aforementioned process. The foundations of the tagging and capture hypothesis are based on the following three major points.

*(i) Protein Synthesis Dependency.* The weak stimulus that induces short-term plasticity phenomena can set tags but cannot synthesize proteins. Nevertheless these tags are able to capture PRPs if they are induced by a strong independent event occurring around the stimulus. So, the administration of protein-synthesis inhibitors impairs the lasting plasticity processes selectively related to the activated inputs. 

*(ii) Temporal Constrains.* Both tag and PRPs have a transient duration. It was shown that if PRPs arrive when the tag has already decayed the capture mechanism did not work. Thus, there is a critical time window of efficacy for tagging and capture process to occur; the order in which tag and PRPs are induced is indistinct, but their temporal coexistence is essential. When Frey and Morris [23] postulated the STC hypothesis and described the first physiological properties of the synaptic tag, they showed that its setting was independent of protein synthesis and that the tag had a limited duration. Therefore, tag and PRPs dynamics limit the time course of the STC process. Since both tag and PRPs have a transient duration, 30 min the first and 1 up to 2 hours the second, there are temporal constraints to the process [25]. Nevertheless, it should be noted that the duration of the coincidence window could be extended or reduced by other processes such as the regulatory mechanisms that accelerate or delay the turnover of synaptic tags and PRPs. 

*(iii) Spatial Constrains.* For the capture process purpose, tags and PRPs should be present at the same neural substrate and at the correct time. If PRPs are synthesized and delivered far away from the point where tags were (or will be) established, the promoting mechanism should be disrupted.

**Figure 1:**
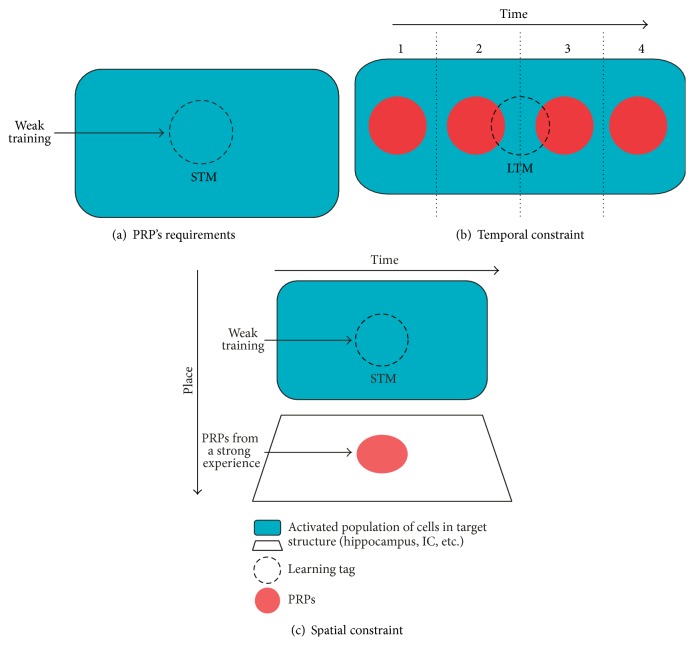
Requirements of the behavioral tagging and capture processes. (a) A weak training that only induces transient form of memory (STM) also induces a learning tag (dashed circle). (b) In order to establish long-term memory (LTM) the tag set by the weak training captures PRPs (red circle) synthesized by an independent strong experience. The process presents temporal constrains and only is effective within a critical time window (only PRPs from the strong events experienced at time 2 and time 3 interact with the learning tag). Note that it exhibits symmetry because PRPs can be captured either if they are synthesized before or after the setting of the tag. (c) The spatial constraint is another important condition that operates in the behavioral tagging (BT) process because the PRPs should interact with the tags; thus, both training events should activate an overlapped population of neurons in the target structure (see (b)). When the learning tag is induced by a weak training (in light blue target structure) in different places where PRPs are synthesized (in white target structure), no BT process occurs and no LTM is observed.

The tagging and capture hypothesis and its dynamics provide an elegant theoretical framework to explain how lasting plastic changes, including LTM formation, occur in the brain. This led us to propose that learning induces the activation of some specific sets of synapses in the network and that in turn this activation could establish a mark (“learning tag”) capable of determining the place where the PRPs should be used and for what they should be used. The BT hypothesis postulates that a learning that induces LTM formation triggers both the setting of a learning tag and the induction of PRPs. To test this assumption the possibility of splitting these processes by using two different tasks was explored. In that sense, a weak-learning task that only induces STM does not cross through the consolidation phase and therefore removes the synthesis of PRPs from the scenario for this task (Figure 1(a)). Then, if the behavioral tagging and capture process exists, the learning tag set by a weak training could use the PRPs induced by the associated task leading to the consolidation of the transient memory into LTM (Figure 1(b)). In agreement with the synaptic plasticity model of STC, in order to capture the products, tags and PRPs should be present at the same time (Figure 1(b)) and at the same neural substrate (Figure 1(c)). Also, the process will exhibit symmetry and PRPs can be captured either if they are synthesized before or after the setting of the tag.

Therefore, if BT process underlies LTM formation, a series of predictions arise as follows.

- BT process should be evident across a diversity of learning and memory paradigms.
- BT process requires setting of tags and availability of PRPs. Thus, blocking one or both of these processes will induce LTM amnesia.
- If tags do not coincide (temporally or spatially) with the PRPs, LTM will not be formed.
- Tags set by different tasks and located in a common population of neurons could compete for capturing available PRPs. Under limited amount of PRPs the competition will be evidenced by the expression of the prevailing memory trace.
- In contrast, sufficient amount of PRPs could induce a more robust and/or persistent LTM trace.

**Figure 2:**
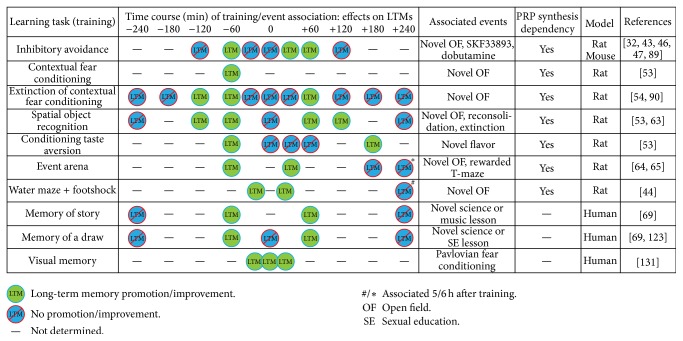
The behavioral tagging process in different learning tasks and animal models. The figure resumes the effects on LTM for different learning tasks associated to another event (associated event) at different time relative to the training session. LTM was generally measured 24 h after training, and it could be promoted/improved, or not. It also shows whether the effect on LTM was reported to be dependent on new protein synthesis, the animal models where the research was conducted, and all the behavioral and pharmacological interventions used as associated event.

These predictions were tested in different learning and memory tasks or activities performed in rodents and humans, and the results are enumerated in the following sections and summarized in Figure 2. Moreover, the BT hypothesis comprised a wide theoretical framework that led us to explain many other questions about memory processing. So other predictions derived from this hypothesis deserve investigation and some of them will be mentioned in the concluding remarks section.

## 3. Time-Related Requirements and Protein Synthesis Dependency for Behavioral Tagging Processes across Different Learning Tasks

### 3.1. Hippocampus-Dependent Associative Tasks

The very first demonstration that LTM formation relays on a behavioral tagging process was reported using a hippocampus-dependent associative learning task in rats: the Inhibitory Avoidance (IA). This was first achieved by developing an experimental design that combined a weak IA training, unable to induce a protein synthesis dependent IA-LTM, with a 5 min novel Open Field (OF) exposure [32]. The rationale involves the notion that OF exposure induces the synthesis of PRPs that should be used by the weak IA learning.

The IA is a versatile hippocampus-dependent and operant-like associative task, in which animals are placed in a box with a platform on the left end of a series of metal bars in the floor and learn that stepping down from this platform results in a footshock. If animals remember this experience, when they are faced again to the platform an increase of the latency to step down is observed. The increase in the test session latency is considered as an indicator of memory formation, being a longer latency indicative of a better memory [33]. This task was advantageous to start seeking a BT process due to two of its main intrinsic characteristics. First, it is a task that can lead to memory formation after a single training session of approximately 10 s. Thus, the processes leading to setting a learning tag and/or the synthesis of PRPs are triggered by a brief and defined training session, in contrast to multi-trial learning tasks where the acquisition, retrieval, and relearning processes occur simultaneously. Another advantage of this task is that the strength of the training can be easily regulated simply adjusting the intensity and/or the duration of the footshock. For example, a strong training (0.5 mA for 3 sec) can lead to the formation of protein dependent IA-LTM, but if a weak training is performed (0.2 mA for 2 sec) only the protein synthesis independent IA-STM can be observed.

The exploration to a novel OF is a spatial behavioral task that even after a relatively brief training of 5 min is able to induce a protein synthesis-dependent LTM of habituation to the arena. This environmental novelty is associated with the activation of the adrenergic and dopaminergic systems and the increment of phosphorylated c-AMP responsive element-binding protein (pCREB) level in the hippocampus [34, 35]. Indeed prolonged exposures to the arena, leading to a familiarization process and the subsequent lack of novelty, were associated to a decrease in pCREB and PKM*ζ* levels [36, 37]. Moreover, the exploration to a novel arena is able to reinforce early LTP into late forms of plasticity [38–40], pointing directly to the possibility of using this behavioral task as a possible PRP donor for other hippocampus-dependent behavioral tasks.

Thus, the first approach to look for a BT process consisted in training rats under weak conditions (wIA), with the intention to induce the setting of a learning tag, in combination to exposing the animals to a novel OF, with the aim to provide the PRPs required by the IA to consolidate its own memory. To achieve this, different group of animals were exposed to a novel OF for 5 min at several times before or after this weak IA training. While those animals that were only submitted to wIA were unable to form a lasting memory 24 h later, different groups that also explored the OF showed a consistent IA-LTM. This promoting effect triggered by the environmental exploration occurred in a restricted time window of approximately 1 hour around the wIA training and it was not observed if the events were separated by long time lapses. This could be explained in terms of the dynamics of the tag and the PRPs: at the time one of the requirements is available, learning tag or PRPs, the other has already decayed. However, it was a remarkable exclusion for the positive effect when OF exposure was experienced 30 min immediately before posttraining times [32]. In sum, we observed that OF exposure should not be too close to wIA, neither too apart of it. The symmetry, manifested by the promotion of IA-LTM when the OF was explored before or after training put into manifest three important things: first, it could not be due to alterations in the conditions of IA-acquisition neither to sensitization processes; second, IA learning tag seems to last for approximately 1 hour and PRPs seem to be available to be captured also during a similar time period; finally, novelty experienced too close around learning might have negative effect that could be attributable to the interference or the resetting of the learning tag. One thing that supports this assumption is the inability to prevent the absence of IA-LTM expression by a further different novel OF exposure given at an effective time [41]. In such case proteins would be still available but the tag would not, impairing the capture process and the consolidation of memory. Consistent with this assumption, it has been demonstrated that a short theta frequency stimulation that resembles neural activity observed in rats exploring a novel environment, when given close to the induction of LTP, can negatively affect the setting of this tag [26, 27, 42].

One important requirement in BT process implies that the learning tag is able to use PRPs provided by the associated experience to allow the consolidation of a memory. Thus, it was essential to analyze if the promoting mechanism leading to IA-LTM was dependent on the synthesis of PRPs triggered by the OF. As predicted, the protein synthesis inhibitor anisomycin infused into the dorsal hippocampus immediately after the OF exposure impaired the promoting effect of the novelty on IA-LTM formation [32]. Moreover, when a single wIA training is combined with two different novel OF explorations (each of them given at a time point that is effective to promote IA-LTM formation), a stronger IA-LTM is formed [43]. This result suggests that, with an extra supply of PRPs, a more robust IA-LTM is expressed. And moreover, blocking the synthesis of PRPs, induced by the second novel OF, through the infusion of anisomycin, resulted in IA-LTM levels comparable to those promoted by the sole exploration of the first novel arena [41].

Proteins induced by novelty are also important to prevent amnesic behavior. PRPs induced by OF exposure rescued the amnesia caused by anisomycin close to a strong training (sIA, which typically induces a lasting IA memory). Moreover, further infusion of anisomycin after the OF session resulted in the expression of IA-LTM amnesia [32]. A similar strategy was applied but using the water-maze paradigm and the results obtained were in accordance with ours [44]. Thus, the novelty preventive effect, as well as its promoting effect, is particularly dependent on its capacity to provide the PRPs required consolidating the IA memory [32]. However, it was recently reported that IA-LTM amnesia induced by scopolamine could be prevented by OF exposure but through protein synthesis independent mechanism [45].

Another interesting aspect of the promoting effect relied on the novel nature of the arena. We have observed that, unlike the exploration of a novel arena, the exploration of a familiar OF, which had already been seen for 30 min in the previous day, is unable to promote IA-LTM [32]. Similar results were observed studying the STC process through behavioral reinforcement of LTP, where exposure to a novel but not a familiar OF was able to reinforce early- into late-LTP [38, 39].

The BT process has then been observed in other variations of the IA step-down learning task. Lu and collaborators [46] showed the first evidence of a BT process operating in mice memory using a step-through IA, in which the animals learn that crossing a door from one compartment to another results in a footshock. They showed that OF exposure was able to promote IA-LTM when performed 60 min before a wIA training, which usually induced STM, and proposed TrKB receptor as a possible tag component. Using the same avoidance task but in rats, Dong and coworkers [47] presented evidence that also the exploration of novel but not familiar objects performed 60 min before a wIA training was able to promote IA-LTM. They showed that this occurs through a mechanism dependent on GluN2B subunit of the NMDA glutamate receptors and LTD.

The contextual fear conditioning (CFC) is another aversive hippocampus-dependent learning task in which LTM has been shown to be processed through a tagging and capture mechanism. In this task, in contrast to avoidance learning, there is nothing that the animals could do in order to avoid punishment. The rodents are placed into a box with metallic bars and, after a brief phase of habituation to the environment, a consecutive series of foot shocks is applied during a certain period of time. The animals are faced to a classical conditioning, in which the simple fact of being in a particular environment is associated with receiving a shock. This association leads to the formation of a usually called fear-driven memory that can be evaluated by comparing the amount of freezing behavior during the habituation period and the test session [48–52]. An increase in freezing is taken as an indicative of memory formation. Similar to IA observation, when the training is performed with weak shock only short forms of memory can be induced, but if this training is associated with the exploration to a novel arena 60 min before, CFC-STM can be reinforced into a CFC-LTM through a mechanism dependent on PRPs synthesis triggered by the novel experience. Thus, both operant and classic conditioning lead to the formation of LTMs through a tagging and capture processes [53]. A nice series of experiments performed by de Carvalho Myskiw and colleagues with this task [54] demonstrated that extinction of CFC memory might also relay on a behavioral tagging mechanism. They have shown that exploration of a novel arena promotes the long-term extinction of the CFC memory through a process dependent on gene transcription and de novo protein synthesis. This promoting effect induced by OF exposure occurs within a critical time window between 2 h before and 1 h after the extinction session. The authors propose that the extinction session is able to set a tag capable of using OF synthesized PRPs in order to induce long-term extinction, and the time course evidences that the CFC-tag of extinction lasts 1 h but is absent after 2 h. As extinction is considered the construction of a new association and therefore a new memory that overcomes the expression of original mnemonic trace [55–57], these results show the other face of the BT process acting in LTM formation.

### 3.2. Hippocampus-Dependent Spatial Tasks

Spatial memories play a central role in our life, they allow us to find or avoid particular places and things by encoding an internal representation of the world. In this sense, the hippocampus is especially important for combining information from multiple sources, as it is required in certain spatial memory tasks [58]. This region that includes the CA fields, dentate gyrus, and subicular complex is part of a system of anatomically related structures in the medial temporal lobe, which is important for many aspects of mammalian memory and for the processing spatial information [59, 60].

The first evidence of a BT process acting in the formation of spatial LTMs came from experiments performed in the spatial version of the object recognition task (SOR). This model can be considered as the rodent version of a what/where memory task, in which the animals should recognize a change in the relative position of two objects [61, 62]. It consists of letting animals investigate an arena with two identical objects for a certain period. Then, in a further test session, one of the objects is changed from its original position and the animals are allowed to explore them again. As rodents display an innate tendency to explore novel situations, an increase in the exploration time of the object placed in the novel position is considered as an indicator of memory. Using this task, it has been shown that a weak training able to induce only STM could result in a lasting memory when it was associated to the exploration of a novel OF. The OF exposure effectively promoted SOR memory within a critical time window that extended from 1 h before to 2 h after the wSOR training, through a mechanism dependent on newly synthesized PRPs. This temporal schedule suggests that SOR task, whose training lasts four minutes, sets a tag that lasts at least 2 h and is completely unable to capture PRPs 3 h after its establishment. Similar to that observed in the IA task, the promoting effect on SOR-LTM was dependent on the novel nature of the arena and it was not observed when the exploration was done too close to the SOR training [53]. Cassini and colleagues [63] have observed that this memory can be also promoted by a quite different source of PRPs. They showed that the protein synthesis dependent reconsolidation process of either CFC or water-maze (WM) learning tasks can promote SOR memory (1 but not 4 h before or after a wSOR training). This promotion was observed only when lasting reconsolidation (CFC or WM) or extinction (CFC) sessions were associated to wSOR training, being the promoting effect also abolished by the infusion of anisomycin in the hippocampus.

Using a completely different spatial memory task, Wang and collaborators [64] provided further evidence of the BT processes underlying the formation of lasting spatial memories. Rats trained in an event arena during several months learned to find a food reward hidden in sand-wells. After that, submitting the rats to a strong-encoding session, consisting of finding a reward of 3 hidden pellets, induced a 24 h memory for the task. On the contrary, a 1-pellet reward encoding session allowed the animals to remember the rewarded location for 30 min but not for a day. However, this weak encoding training could lead to a LTM if the training was associated to the exploration of a novel OF. In coincidence with the previous observations, this promotion was symmetrical, dependent on the novel nature of the arena and on the synthesis of new PRPs induced by it. Using this appetitive-driven spatial memory Richard Morris group showed that, as well as in single trial learning experiences, encoding and storage of an everyday learning-like experience can lead to memory consolidation through a BT process. More recently it has been shown that not only spatial novelty but also a rewarding experience in the T-maze was able to promote this particular memory when experienced 30 min but not 3 h after training. On the contrary, a novel object recognition task was unable to promote this memory, putting into evidence that different tasks are able to promote event arena-LTM but not all novel experiences act in this way [65].

Implementing an unusual experimental approach, Almaguer-Melian and coworkers [44] have shown that the WM memory could be also processed through a BT mechanism. In the WM learning task rodents are intended to remember the location of a hidden escape platform placed into a small pool of water (with visual cues). When first released in the pool, rats swim around searching for an exit until they eventually find the platform. A decrease in the time (latency) that takes finding the platform in the successive sessions is used as an indicator of memory. The authors showed that four trials in the WM were sufficient for rats to remember the location of the platform at the following day. Interestingly, if the animals were submitted to a foot shock (FS) session performed after training, the consolidation of the WM-LTM trace was impaired. In resemblance to the other results in hippocampus-dependent learning tasks, when animals were also submitted to an OF exploration 15 minutes before or after WM training, the memory was recovered in a protein synthesis-dependent way, overcoming the disruptive action of the FS on WM-LTM formation [44]. This recovery effect occurred during the first moments around training but not if the OF exposure was performed 4 hours apart from training, showing that it is a time dependent process. It is worth mentioning that as WM-LTM could be recovered by the novelty induced PRPs, this strongly suggests that FS did not interfere with the setting neither the maintenance of the WM-learning tags. Therefore, a tempting explanation is that massive neuronal activation triggered by the strong FS depletes the system from the available PRPs, causing a long-term WM-amnesia that can be reverted by providing extra proteins from an external source like novelty.

### 3.3. Cortex-Dependent Associative Task

So far, we have shown consistent evidence of the BT process acting in the formation of several qualitatively different LTM that have the common characteristic of being processed in the hippocampus. Nevertheless, neither the hippocampus is the only region involved in the formation of lasting memories, nor all memories are processed solely in the hippocampus. Thus, it is essential to investigate if the BT process acts in the formation of lasting memories processed in other brain structures.

Nowadays, this evidence comes from experiments performed in the conditioning taste aversion (CTA), a learning task processed in the insular cortex [66, 67]. In our experimental conditions, this task seems to be hippocampus-independent; however, a recent work using conditional knock-out rodents lacking the hippocampal NMDA receptor-NR1 subunit found an opposite result [68]. Taste-recognition memory is part of the essential spectrum of skills that many animals require to survive. In wild life, remembering whether a particular taste or flavor is associated with a malaise by intoxication or poisoning, is essential for the survival of the animals. The CTA model of memory allows animals to associate a specific flavor with a digestive disorder. During training session animals with restricted access to water are submitted to consume either water or saccharine sweetened-water and after 30 min. Those animals that taste the sweet water are then intraperitoneally injected with saline or a lithium chloride solution. This substance causes an intensive digestive malaise and therefore a decrease in the consumption of the flavored water during the test session, which is taken as an index of memory. Rats that received a low dose of LiCl (weak training) induced a negligible CTA-LTM but expressed a strong CTA-STM measured 30 minutes after the acquisition session [53]. In order to analyze a BT process in this memory, a PRP donor had to be found. Thus, a new strong flavor (NaCl) was instrumented as novel insular dependent experience. As a result, the association of a weak CTA training with the consumption of a NaCl solution, 1 hour before or 2.5 hours after the training, but not in between them, resulted in a robust CTA-LTM. So, the CTA learning may involve longer processing time to be set because the process requires the association between two stimuli that are distant in time (the ingestion of saccharin and the effect of the lithium chloride injection). In agreement with this idea, there is no promotion observed in the time window between 0 and 1 h after training but there is promotion at 1 h before and at 2.5 h after learning, showing that tag setting might be interfered by events close to training and that the tag remains functional long time after saccharine consumption. In accordance with the observations performed in the different studied hippocampus-dependent tasks, the promoting effect of novelty in CTA also depends on both the synthesis of new PRPs induced by the consumption of NaCl and the novel nature of this flavor, as animals familiarized to this taste did not present any improvement in saccharine CTA-LTM [53].

Considering the whole data included in this section, the BT process seems to be a general mechanism for consolidation of aversive, spatial, extinction, hippocampus-dependent, and cerebral cortex-dependent memories. The characteristics of the process determine the complexity of the temporal requirements which resolves whether different behavioral events interact positively or negatively, depending on their intrinsic features and their temporal separation. The third important requirement of the BT process regarding the overlapping of the neuronal substrate activated by interacting tasks will be discussed below.

## 4. Spatial Constrains and Specificity of the BT Process

Thought as a behavioral analogue of the STC, the BTC process must occur within a critical time window and is restricted to the tagged substrate. This does not represent a problem when the proper learning experience can induce the learning tag and also the PRPs; however, when a weak task is associated to a strong PRPs donor experience, the processes triggered by both of them should be integrated in the same neuronal substrate or at least in overlapped neuronal networks. For this reason all the experimental designs used to study the BT rely on tasks processed, at least partially, in the same brain structures. This gives strong support to the idea of spatial restrictions of the BT process. But in the absence of microscopy data showing the coactivation of overlapped neuronal networks, the best control of the spatial requirements of the BT process untill day comes from a set of behavioral experiments. They show that a novel experience capable of providing PRPs but processed in different brain areas than those capable of inducing learning tags were not able to promote lasting memories. Based on the fact that a taste recognition memory is mainly processed in the insular cortex and that spatial learning is strongly dependent on the hippocampus, we explored the possibility to promote the formation of CTA-LTM through a novel OF exposure and reciprocally promote SOR memory through a novel taste. In this case we observed that neither the exploration of a novel OF 1 h before or 2.5 h after a wCTA training nor tasting a novel smack 1 h before or after a wSOR training (permissive time points in which novel taste promoted CTA memory and novel OF SOR-LTM) was able to promote the consolidation of these memories. Therefore, neither the hippocampus-dependent task was able to promote an insular cortex-dependent memory nor an insular cortex-dependent task was able to promote a hippocampus-dependent one, putting into evidence that spatial coexistence of tags and PRPs must occur in order to allow the consolidation of a lasting memory [53].

The other spatial constraint of the BT process is related to the concept of input specificity. In other words, PRPs are supposed to be captured only by the tagged sites, reinforcing only these sites and not all the available inputs of the network. This concept was evaluated through a behavioral approach submitting rat to a wSOR training followed 3 h latter by a second training that involved a different pair of objects. An OF exposure experience one hour before the first wSOR training session promoted the SOR-LTM only for the pair of objects explored during this training but not for those explored 4 h latter [53]. These findings indicate that BT displays input specificity, allowing LTM formation for the learning that sets learning tags during a permissive time in which novelty promotes spatial memories. Moreover, similar results were observed in school children, where a novel science lesson experienced 1 or 4 h after two different stories told by their teachers were able to specifically improve the memory for elements of the story listened 1 h before the novel experience [69].

## 5. Memory Competence: Another Aspect of BT Process

The BT hypothesis displays wide scenery where the tag set by different learning experiences could interact with the PRPs present at those places in a given time. In the aforementioned cases, a weak learning was beneficiated by using PRPs derived from a strong experience. However, what would happen if the number of tags is larger than the available PRPs supply? It is reasonable to predict that one of the LTM traces will be negatively affected when the protein supply was insufficient to maintain memory processing of both tasks.

Evidence from LTP experiments shows that under regimes of limited protein synthesis two potentiated pathways can compete for protein resources needed for the establishment of L-LTP [70]. In this case, when a weak and a strong tetanizing stimuli were applied simultaneously, LTP was maintained for hours at both inputs. However, applying a further weak tetanus in the presence of anisomycin resulted in the potentiation of the reactivated pathway at the expense of the persistence of LTP on the other. Recently, by stimulating three different pathways around the same time, a “winner-take-all” process was defined and the temporal dynamics for the competition between these three plasticity events was described. The authors showed that when the L-LTP was enabled on one-weak stimulated pathway by virtue of the utilization of PRPs induced by another closed event, potentiation of a further third pathway around the same time might prevent persistent potentiation on all pathways [71]. Furthermore, stimulated synapses would compete for limiting PRPs synthesized at the dendrite compartment for the establishment of LTP. Thus, the stimulation of multiple inputs within a short distance resulted in the growth of one spine, accompanied by the shrinking of the others [72].

**Figure 3:**
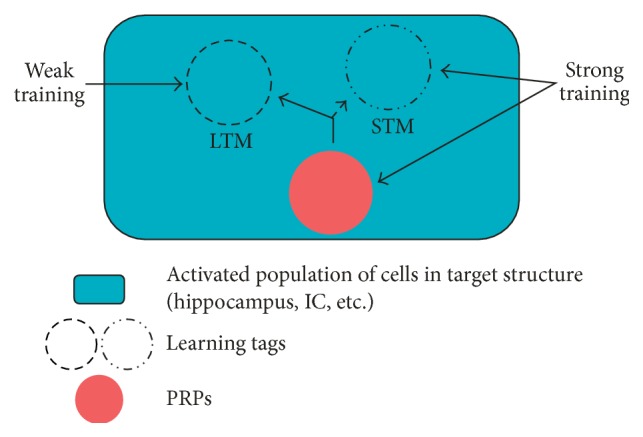
Competence for PRPs in LTM formation. A strong training not only triggers the synthesis of PRPs (red circle) but also induces its learning tag (dashed-dotted circle). Weak training experienced close to the strong one only set its corresponding learning tag (dashed-circle). So, these different types of tags could compete inside a cell to capture the PRPs that are available around them. If the amount of proteins is limited it was observed that one LTM is promoted and the other one is impaired. This process should be accomplished in a subset of cells which were activated by both training experiences.

We hypothesize that if different learning experiences are being consolidated into LTM, intracellular competition for PRPs among their respective learning tags will define which of the memory traces becomes stabilized in the neuronal network. Based on the protocols of the first BT experiments [32], this has been tested by combining wIA and novel OF, two tasks that are dependent on hippocampus processing. If rats are sequentially exposed to two different memory tasks under limited protein synthesis, OF exploration promotes IA-LTM formation from a wIA training session and this occurs in detriment of the OF's LTM [43] (Figure 3). In contrast, but in accordance with the time window of efficacy to the promoting effect of novelty, when tasks were separated by a larger time lapse, LTM of habituation was present. We also demonstrated that when subjects are trained in a wIA and explore two different and novel OF arenas (1 h before and 15 min after wIA training), IA-LTM is further improved. In parallel, whereas LTM for the first OF is impaired when wIA is intercalated between both exploratory sessions, the second OF-LTM was preserved [43]. In such scenario, we concluded that the levels of PRPs may be insufficient to satisfy the LTM requirements of the three behavioral tasks and thus not all mnemonic traces would be consolidated.

But which are the PRPs acting in this process? Activity-regulated cytoskeletal associated protein (Arc) has been shown to be involved in the formation of several types of memories and has an important role in synaptic plasticity [73–75]. In particular, the use of Arc mRNA antisense oligonucleotides, delivered into the dorsal hippocampus after a novel OF exploration session, was shown to have deleterious effects in novelty promoted IA-LTM formation. This fact suggests that Arc is required for both types of memory and their learning tags which competed for it [43]. Latest research suggests that Arc is captured by CaMKII*β*, which induces an “inverse synaptic tagging process,” recruiting Arc in the less active terminals. Arc, in turn, downregulates the amount of GluA1 at individual synapses [76]. Even though Arc is an attractive candidate as a PRP that could be disputed between memory traces, other PRPs related to plastic changes in synaptic terminals should be considered as well [77].

These results lead us to think that competition for protein resources between different learning tags is one of the main factors that give rise to memory interference. Centenary observations postulate that retrograde interference (RI) in a memory consolidation is exerted by an event experienced after the first learning session and that this RI increases with the proximity between events. Traces become less vulnerable to empirical forgetting, brain damage, or retroactive interference as they consolidate with the passage of time [3, 78–80]. It is suggested that the interpolated event causing RI could be a similar material to be encoded, with the RI being reduced when tasks are highly similar or, on the contrary, when they are markedly different [80]. This could be reinterpreted considering the BT hypothesis, involving the capture of PRPs by different kinds of learning tags. If the interpolated event is identical to the original, it can represent a retraining and reinforces almost the same learning tags set by the original task. Moreover, a high dissimilarity of the events could imply its processing in different brain regions; thus, the respective learning tags would not interfere because they do not show spatial overlapping [53, 81]. In that sense, we recently reported that the RI caused by an object in context training trial, over a previous different one, is supported by an effective temporal window between events and by the regional brain areas involved in the processing of their LTMs. Thus, when the interval intertrail was enlarged as well as when we inactivated the dorsal hippocampus or the mPFC, previous to the second training trail, the RI disappeared [82]. This result strongly suggests that LTM-RI amnesia is probably caused by a lack of resources due to cellular machinery competition in these brain regions when they are engaged in the formation of memory traces.

Based on all data compiled up to now, the BT model proposes a cellular mechanism to explain LTM promotion by novelty-associated event as well as amnesia by interference, focusing on the competitive capture by tags of PRPs required for the consolidation of those memory traces. In sum, the final behavioral outcome will result in accordance with the type of learning tasks, their training conditions, and temporal protocol schedule. The question that emerges here is if there is some experimental evidence about the identity of the tag, the PRPs, or any support for their specific capture. In the next section, we summarize relevant information about these issues.

## 6. Searching Candidates for Tag and PRPs—Cellular Evidences for PRP Recruitment to Tag Sites

There are several criteria to be satisfied by a synaptic tag [23, 83–87]: a tag can be set by different stimulations able to induce early or late forms of synaptic plasticity; the lifetime of a tag is transient during less than 2 h but may be extended by metaplastic influences [88]; the activation of a tag does not require protein synthesis; a tag is induced in an input-specific manner and is relatively immobile; and finally a tag must interact with (and therefore capture) the PRPs to stabilize a late plastic changes. Extending these assumptions to the memory field, a learning tag was also defined, where a behavioral training can induce a kind of mark to signal the place and the critical time where different products could interact to allow LTM formation [32, 86, 89, 90].

A postulate of STC and BT hypotheses is that the tagged sites should capture PRPs in order to establish long-term plasticity or memory, respectively. Some empirical evidences supporting this enunciate will be summarized here. Matsuo et al. [91] developed transgenic mice to monitor the trafficking and turnover of newly synthesized AMPARs induced at the time of learning in a fear conditioning paradigm. These transgenic mice expressed the GluA1 subunit fused to green fluorescent protein under control of the* c-fos* promoter. The results show a preferential recruitment of newly synthesized green GluA1 to mushroom-type spines in adult hippocampal CA1 neurons one day after training, suggesting that the learning induces changes in some spines allowing the capture of PRPs at later time points. The authors conclude that a synaptic tagging mechanism operates during behavioral learning and implicated GluR1-containing AMPARs as one of the cargo molecules selectively delivered to tagged synapses.

Another data supporting the synaptic tagging mechanism consists in input-specific spine entry of Vesl-1S (Homer 1a) protein triggered by the activation of N-methyl-D-aspartate (NMDA) receptor [92]. The authors trace the transport of an EGFP-fused form of Vesl-1S, which is synthesized in soma, into the dendritic spines of rat hippocampal neurons in primary cultures. They observed that the green protein stayed in dendrites unless the NMDA receptor was locally stimulated in the spines. This activity-dependent trapping was independent of protein synthesis. On the basis of these results, the authors propose that Vesl-1S protein is a PRP that behaves in a manner consistent with the synaptic tagging hypothesis.

Capture/utilization of PRPs at tagged sites enables lasting changes in the synaptic efficacy at those sites, allowing L-LTP stabilization after a weak stimulation that normally induces e-LTP. This was usually measured as the average response of a population of stimulated synapses [23, 93]. To demonstrate this phenomenon at single-synapse level, Tonegawa's lab developed a method that permits local stimulations. They used two-photon glutamate uncaging at single spines on dendritic branches of CA1 pyramidal neurons and monitored the spine volume change as a measure of both L-LTP and e-LTP [72]. They studied how L-LTP induction at a given spine affected other spines. A STC mechanism was observed when a strongly stimulated spine facilitated induction of L-LTP at a neighboring weakly stimulated spine. This phenomenon was dependent on protein synthesis induced by strong stimulated spine because no growth was seen at any spine if protein synthesis was blocked throughout the experiment using either anisomycin or cycloheximide. They found that STC is temporally asymmetric, spatially localized, and biased toward stimulated spines that reside on the same dendritic branch. The authors proposed a model named the Clustered Plasticity Hypothesis where the capture of protein is favored for closer synapses in a dendritic branch and there is less protein available to synapses farther away [72].

Although the classical STC theory proposes a cell body-initiated gene expression in order to provide PRPs, a local protein synthesis at individual synapses is also possible. In that sense, a reductionist model termed “synaptic sushi belt” [94] unifies these alternatives by means of ribonucleoprotein particles (RNP) transport by motor proteins along the cytoskeleton leading the diffusion and trapping by a localized anchor. They postulate a constant bidirectional transport of RNPs in dendrites of mature neurons, being only the synapses that have been previously activated (tagged) able to capture this RNP from the cell body allowing the local translation of specific transcripts.

In the following, we will describe the molecular and cellular data obtained for tags and PRPs in behavioral models of learning and memory.

## 7. Mechanisms Involved in Learning Tag Setting and PRP Synthesis in BT Process

The BT hypothesis relies on a mechanism composed of two complementary processes: the setting of a learning tag and the synthesis of those PRPs that once captured by the tag will allow the storage of a memory for long periods of time. However, a learning leading to LTM formation initiates both processes simultaneously. Thus, these processes could be studied and evidenced only by dissecting them through a dual task BT protocol. The methodological approach relies on the local infusion of drugs around the time of learning task or around the time of the event that induces PRPs synthesis. Therefore, any intervention capable of disrupting the learning tag should result in an irreparable amnesia. However, amnesia caused by interference to the synthesis of PRPs should be prevented or rescued by providing external PRPs suitable to be captured by tag. Here, we summarized data concerning the mechanisms underlying these complementary processes.

A common characteristic of the tagging and capture research relies on the fact that most of the strong events that served as PRPs providers to promote lasting memories or that reinforced synaptic plasticity had the singularity of being either a novel experience or a familiar experience with a novel component [32, 38, 39, 44, 47, 53, 54, 64]. Novelty detection has been consistently linked in many ways to the activation of the ventral tegmental area (VTA) and the locus coeruleus (LC), as well as to the release of dopamine and adrenaline in several brain structures [95–99]. In turn, dopaminergic and adrenergic receptors activation triggers different second messenger cascade that can result in gene transcription and eventual translation process. Thus, it is not surprising that these systems were the first to be studied as candidates for controlling the synthesis of PRPs triggered by novelty. This dopaminergic dependence was first reported in functional plasticity. Both Li and colleagues [38] and Straube and collaborators [39] observed that antagonizing the dopaminergic receptors in the hippocampus at the moment of a novel experience completely impaired the novelty-dependent reinforcement of e-LTP into L-LTP. First behavioral clues were provided by Moncada and Viola [32] working with the IA task. This work showed that the promotion of IA-LTM, triggered by the exploration of a novel OF 1 h before a wIA training, was impaired by the infusion of the D1/D5-dopaminergic receptor antagonist SCH23390 (SCH) in the dorsal hippocampus 10 min before the novelty session. Years later, similar results were obtained when the *β*-adrenergic antagonist propranolol was infused into the dorsal hippocampus [89]. Moreover, dopamine receptors were also required in the BT models based on the schemas and WM memories, in which antagonizing D1/D5-dopaminergic receptors during the associated novel experience impaired the promoting effect over the schemas-LTM and the novelty dependent prevention of the stress-induced amnesia over the WM-LTM [44, 64].

Further evidence, supporting that these receptors may be responsible for triggering the synthesis of PRPs, came from experiments using a mimicking strategy instead of a blocking strategy. In this case, the replacement of the novel experience by intraperitoneal administration of dopaminergic (SKF 38393) or adrenergic (dobutamine) agonists before a wIA training leads to a promotion of IA-LTM. This promoting influence was observed when drugs were injected 70 but not 180 min before training, showing a time windows of efficacy remarkably consistent to that observed when novelty was used as memory promoter. Moreover, the effect was completely blocked by the infusion of anisomycin or emetine into the dorsal hippocampus, showing that SKF 38393 and dobutamine promote IA-LTM through a protein synthesis dependent mechanism that occurs there where memory information is being processed [89].

Taking into consideration that memories require their particular set of proteins to be consolidated, the previous series of experiments encouraged us to think that those mechanisms used by novelty to promote memory could be the same mechanism triggered by a strong training capable of inducing lasting memories. This issue was analyzed for the IA task by studying the role of D1/D5-dopaminergic and *β*-adrenergic during strong trainings induced LTM. Here it was shown that IA-LTM induced by sIA training was completely blocked if learning session occurred 10 min after the infusion of either SCH23390 or propranolol in the hippocampus. Interestingly, this amnesia was prevented when animals were let to explore a novel OF 60 min before the sIA training. The fact that this amnesia could be reverted by the exploration to a novel arena shows that neither dopaminergic nor adrenergic receptors were involved in the setting of the IA-learning tag; but on the contrary, they should be affecting the synthesis of PRPs triggered by the strong training. Moreover, further infusion of anisomycin after novel OF impaired its preventive effect [89]. Indeed, the protein dependence of the preventive effect puts into evidence that the activation of D1/D5-dopaminergic and *β*-adrenergic receptors, in the hippocampus during sIA training, is specifically involved in the regulation of the synthesis of those PRPs required for the consolidation of this memory. Recent research, presented at the Neuroscience Forum, also showed that VTA and LC activation are responsible for controlling the consolidation of the IA and SOR memories by regulating the synthesis of PRPs in the hippocampus [100]. Thinking in these structures as responsible for controlling the synthesis of PRPs may explain how the alteration in dopaminergic and adrenergic systems around the learning of different tasks results either in the impairment, modulation, or promotion of lasting memories. A total absence of PRPs would induce amnesia, providing PRPs to a learning experience unable to induce their synthesis which would result in promotion of that memory, and finally, modulation in protein expression should result in the formation of better or worst LTMs.

In contrast to these catecholaminergic receptors, which have been shown to be required in the hippocampus for the formation of LTMs but not of STM, NMDA glutamate receptors resulted to be essential for both of them [89]. Moreover, the infusion of NMDA receptor antagonist AP-V in the dorsal hippocampus before a weak or strong IA training resulted in the absence of IA-LTM even when animals had been also submitted to explore a novel arena 60 min before IA learning session [89]. As PRPs synthesized by action of novelty were available to be used by IA-learning tags to allow memory consolidation, the absence of IA-LTM shows that NMDA receptors activity is essential for the setting of the IA learning tags. Similar results were obtained by Cassini and colleagues [63] who have shown that the promotion of SOR-LTM, by a CFC reconsolidation event, was completely impaired by the blocking NMDA receptors before SOR training. Beyond the novel role of NMDA receptors in the setting of a learning tag, there is an extensive body of evidence showing that their activation triggers different signal transduction processes leading to the synthesis of proteins that can be used during consolidation [33, 101–103]. Thus, we think that NMDA receptors play a dual role being responsible for triggering events that set the learning tag and events that induce the synthesis of PRPs. Actually, BT experiments in which the local infusion of AP-V, previous to a novel OF session, impaired the usual promotion of a lasting IA-memory suggest the involvement of NMDA receptor in the protein synthesis process [89].

NMDA receptors might be essential for the learning tag, but they are not the tag itself. Indeed, either at functional plasticity or behavioral level, the tag is considered an ensemble of molecules tending to modify the morphology of the dendrite [87, 104, 105]. Thus, it is reasonable to think in NMDA receptors as one of the first echelons of the tagging machinery. But the complete configuration of the tag is an enigma that started to be studied at synaptic and behavioral level since the first moments in which the theories were postulated. In that sense, protein kinases were always interesting targets of research due to their fast activation and to their speed in modifying the response of receptors and structural morphology of the spines. Some particular kinases such as *α*CAMKII, PKA, and ERK1/2 are involved in the formation of LTM since the very first moments after learning, making them interesting candidates as tag components [5, 33]. Their specific role in the BT process was studied using a wIA training in two tasks experimental design. There, the hippocampal infusion of KN-62 (*α*CAMKII inhibitor) or Rp-AMPc (PKA inhibitor) 10 min before or 15 min after wIA, but not 1 h after it, impaired IA-LTM promoted by the exposure to a novel arena 1 h before training [89]. A third kinase, PKM*ζ*, resulted to be partially necessary in the very initial moments of the tag setting but was shown to be required even 1 h after wIA training [41]. In contrast, neither U0126 (MEK inhibitor) nor anisomycin was able to impair the promoting effect of novelty when infused into the dorsal hippocampus close to a wIA training. These results suggest that *α*CAMKII, PKA, and PKM*ζ* play an essential role in the setting of the IA learning tag, while its machinery does not require the activity of ERKs 1/2 neither the synthesis of further proteins [89]. Additional information of the learning tag machinery came from experiments performed with TrkB knock-in mice in the step through IA task. Lu and collaborators [46] demonstrated that inhibition of this receptor's kinase activity, during a weak training, also impaired the promotion process induced by novelty. In the same work they presented analogue* in vitro* experiments, showing that TrkB inhibition during a weak tetanization protocol also blocked the reinforcing effect of e-LTP into L-LTP by an associated strong tetanization of a confluent path and postulated this receptor as potential component of the learning and synaptic tags [46]. Interestingly, while the setting of IA-learning tag as well as LTP- and LTD-tags is protein synthesis independent processes, recent experiments showed that the tag setting during CFC extinction learning might depend on it [54]. Interestingly, this tagging process seems to be dependent on NMDA and L-VDCCs receptors as well as protein synthesis, through a mechanism that relays on the proteasome ubiquitin-mediated protein turnover [90].

In general, all the components of the tagging machinery in behavior are consistent with those identified in the electrophysiological model of synaptic tagging. Functional plasticity experiments show that CAMKII is specifically required for the setting of the synaptic tag while CAMKIV is recruited in the soma via CAMKK to regulate the synthesis of PRPs [106]. Interestingly *α*CAMKII has been shown to be required for the setting of LTP tags in the apical compartment of the pyramidal cell in CA1 region of the hippocampus, while PKA and PKM*ζ* seem to be responsible for setting the tag at the basal region [28, 106, 107]. The fact that CAMKII and PKA are essential for the setting of the learning tag with the same time requirements but that both could be acting at different neuronal compartments opens the question of whether they are required for processing and storing different aspects of IA memory. The requirement of PKM*ζ* in the BT process presents different dynamics being required even 1 h after wIA learning suggesting that it may be required for a late maintenance of the learning tag. Therefore, this kinase that has been shown to be required for late maintenance of memory and functional plasticity processes [108] could be also required to maintain early plastic changes in the learning tag as well. In this direction, suggestive evidence has shown that PKM*ζ* controls metaplastic changes of the synaptic tag, through the regulation of the trafficking and degradation AMPA receptors, allowing a prolongation of the time in which transient potentiation can be reinforced into L-LTP [31, 109–111]. Up to now, the role of PKM*ζ* in long-term memory and plasticity processes is currently in the center of a debate due to experiments performed with knockout mice, reporting normal learning and possible nonspecific Myr-zip blockade [112, 113] and more recent information showing that PKM*ζ* is compensated in knockout mice and confirming Myr-zip as a potent competitive inhibitor of PKM*ζ* in neurons [114–117]. Nevertheless, we think that the amount of information linking this kinase to different processes and phases of synaptic plasticity and memory formation defines its importance as relevant and viable participant in neuronal representations of memory.

In contrast, ERK1/2 kinases have been shown to be required specifically for the setting of synaptic-tags associated with LTD [107, 118]. Thus, the lack of requirement of ERK kinases for the setting of the IA-tags is consistent with the idea that avoidance memory might be processed by mechanisms associated with LTP induction [13]. Interestingly, recent findings show that exploration of novel objects promotes avoidance memory through a LTD-like process [47]. Thus, a possible processing of spatial novelty through this kind of mechanism, could lead to impairment or resetting of probably LTP-like IA tags.

**Figure 4:**
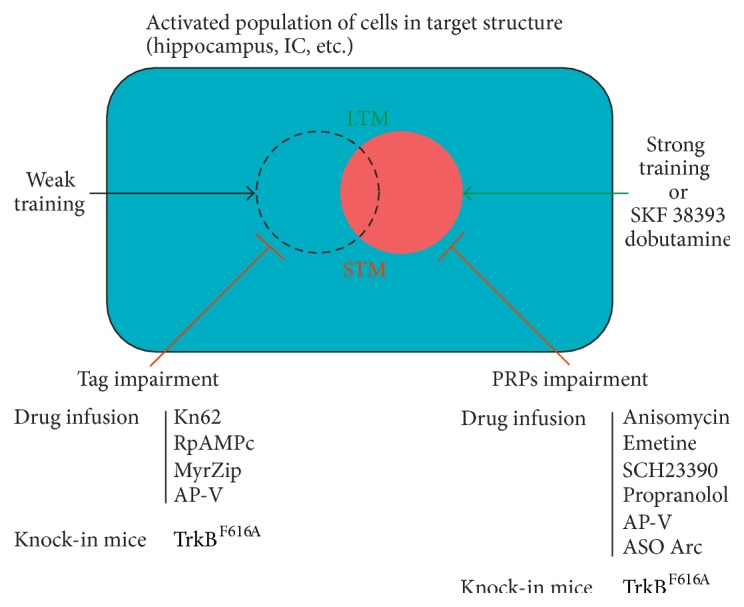
Strategies used to study the processes related to the setting of the learning tag and the PRPs synthesis in BT models. Weak training induces short- but not long-term memory and sets a leaning tag (dashed circle). The strong experience or different drugs (dopaminergic and adrenergic agonists) induces the synthesis of PRPs (red circle) that can be used to allow memory consolidation for a weak learning (green path). The local infusion of different inhibitory drugs (i.e., *α*CAMKII, PKA inhibitors, PKM*ζ* blocker, or NMDA receptor antagonist) in brain structures close to the weak training can interfere with the proper setting and/or maintenance of the learning tag, impairing the promotion of LTM (red path). The local infusion of different drugs (i.e., protein synthesis inhibitors, antisense oligonucleotides, or D1/D5-dopaminergic, b-adrenergic, and NMDA receptors antagonists) in the target structure at the moment of PRPs synthesis also impaired the promotion of LTM (red path). Kinase activity requirement of TrkB receptor for both processes has been shown using Knock-in mice (see [43, 46, 54, 63, 64, 89]).

In sum, up to now the processes leading to the synthesis of PRPs seem to rely on dopaminergic and adrenergic systems, as well as on the requirement of NMDA receptors, with Arc and TrkB being two of the possible PRPs to be captured. On the other site the setting of the learning tag has been shown to be independent of PRPs synthesis and relying on NMDA receptors functionality as well as *α*CAMKII, PKA, PKMz, and TrkB. Most of these mechanisms that were reported using the IA-novel OF BT model but confirmed in schemas and WM spatial memory tasks are summarized in Figure 4. BT machinery has also been shown to be coincident with the mechanisms reported in the STC process for LTP. Nevertheless, a remarkable difference has been shown for the CFC-tag of extinction, which seems to rely on protein synthesis and to be independent of CAMKII activity.

In the next section we will show some evidences of a second round of tagging acting on a late consolidation phase required for late persistence of memory.

## 8. Is There a BT Mechanism Leading to LTM Persistence?

**Figure 5:**
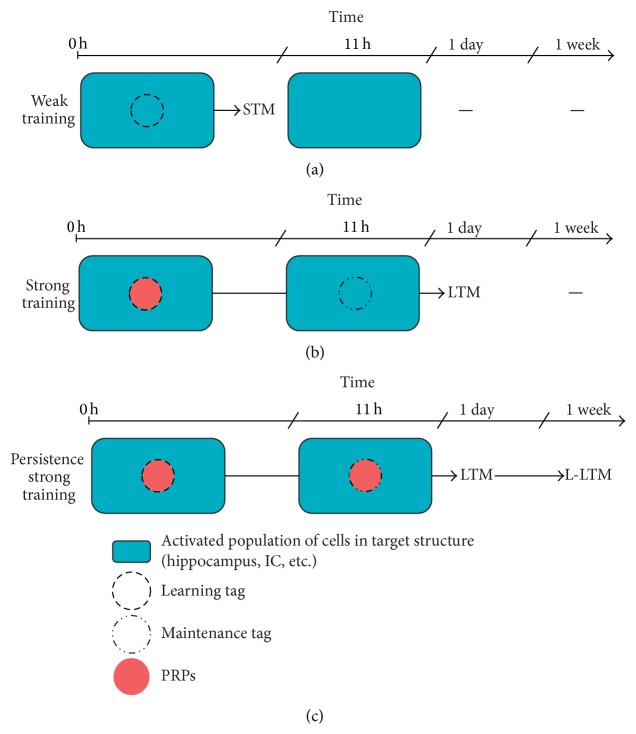
Behavioral tagging in LTM-persistence. In the life of a memory could be at least two rounds of tags. (a) At the time of a weak training, a learning tag is set (dashed circle). However, as there are no PRPs synthesis, consolidation does not occur. (b) A strong training* per-se* triggers two processes: the setting of a learning tag and the synthesis of PRPs (red circle). The capture of these products by the learning tags led the formation of a LTM (usually tested 1 day after training). Moreover, we postulated that the strong training also induces in a delay fashion a second round of tagging (maintenance tag) emerging at 11 h after training (dashed-dotted circle). (c) A stronger training (persistence strong training), will allow memory to persist at least for a week (L-LTM). This persistent expression of memory could depend on the capability of a late established maintenance tag (dashed-dotted circle) to use/capture PRPs (red circle) derived of a late wave of protein synthesis. It was reported that this very strong training session induces a delay window of protein synthesis on which its memory persistence relies. Also, in the case of a strong training session (which induce only LTM), PRPs provided by a pharmacological intervention or by a behavioral experience can promote L-LTM if they are available around the time of the maintenance tag.

It is well known that a late BDNF (brain-derived neurotrophic factor) and protein synthesis dependent phase of memory formation, occurring around 12 h after strong IA training in the dorsal hippocampus, is required for memory persistence [6, 119, 120]. Expanding the postulates of STC hypothesis and its BT translation, we further think that beside the tagging process displayed in memory consolidation, some “retagging” of specific sites would occur late after training enabling memory persistence through the capture of these late PRPs. So, we propose that a learning experience able to induce a LTM could signal at least two marks separated in time (immediate after IA training and 11-12 h later), which capture PRPs to allow, in first instance, the consolidation of a LTM and then to grant its persistence for longer periods of time (Figure 5).

Based on the promoting effect of a novel OF exploration on IA-LTM formation [32] and considering the late protein synthesis window after a strong IA training, we tested if it was possible to promote a persistent memory (named L-LTM, operatively measured 7 days after training) from a IA training that induced a LTM that decay after a couple of days. For that mean, rats were submitted to a novel OF exploration 11 h after a IA training session, which only induces LTM, and IA memory evaluated after 7 days, to analyze whether its persistence was enabled as a result of applying this protocol. Our results strongly suggest that this IA training would create a maintenance-specific tag where PRPs provided by the OF are employed to enable the persistence of the IA memory, resulting in L-LTM [121]. The exposure to a novel OF is effective in the promotion of L-LTM only when novelty occurs around 11 h after IA-training, and it is ineffective outside this temporal window [121]. This strongly suggests that not only the PRPs delivery is important, but it is also essential that the system is prepared (“retagged”) to use the products derived from the novel experience. This effect on memory persistence requires the activation of dopamine D1/D5 receptors and Arc expression in the dorsal hippocampus around OF exploration [121]. In line with these results, it was previously observed that either a stressful event or the administration of corticosterone 12 h after a contextual fear conditioning selectively prolongs the persistence of this LTM [122]. The effects induced by the stress were prevented by systemic administration of metyrapone, a corticosterone synthesis inhibitor.

The idea that a strong learning could set a late but transient “maintenance tag” opens wide scenery where capture as well as competence for PRPs several hours after acquisition affects memory survival. This offers a behavioral strategy to improve or potentially impair the durability of memory traces, helping to memorize some events or to forget some others.

## 9. BT in Human Memory

The consistent amount of evidence supporting that the formation of lasting memories occurs in rodent models through BT mechanisms leads directly to question whether human memories can be established through this process as well. The study of BT in humans has important constrains; in particular, the study of PRP synthesis dependency is not possible. Nevertheless different strategies can be applied to infer such a BT process underlying human memories. A first report supporting this assumption came from activities performed with students of Argentinean elementary schools. By using a similar approach to those previously mentioned, we analyzed the memory for either literary or graphical activities when these were combined or not with novel and familiar experiences. Activities were conducted inside the school and were leaded by the corresponding teachers under our supervision. We observed that certain groups of students that also attended a novel science lesson presented important improvements in LTM for both activities. This effect was observed when the novelty was presented one, but not four hours, before or after the learning lesson and was particularly strong on those components difficult to remember [69, 123]. Similar improvements were observed when the students attended a novel music lesson instead of a science one, but this effect was absent when this lesson was familiar because they had already attended it two times before in the previous weeks. Another interesting property relies on the task's time specificity of this process. When students learnt about two different activities separated for 3 h, instead of merely one, and they attended the novel science lesson 1 h after the second activity, they only presented memory improvements over aspects of the activity closer to the novelty [69]. Overall, these experiments show that a novel pedagogic experience, during regular school time schedule, can improve memory of different activities performed with the students' teacher. Since novelty improves memory also when presented after the activity, this effect cannot be awarded to changes in the attention levels of the students or in the basal conditions of learning, stressing the idea that novelty effects may be acting through a BT mechanism. In that sense, students that observed an emotionally arousing video after a lecture in a psychology course evidenced an enhanced memory two weeks after the experimental manipulation [124]. Also, novelty is efficient to promote LTM when it is experienced before the learning class. In accordance with this view, Schomaker and coworkers [125] found that when people experienced a video novel environment exploration before a word learning task, they had a memory improvement of the words during a free recall phase. Since memory enhancement is found when the novelty arousing experience occurs before and after the learning occurs, this improvement probably cannot derive from an arousal state or from lowering the threshold to learn. We postulate an alternative idea based on the involvement of BT mechanism in these memory processes.

Neurobiological research has recently confirmed that entrance of new information into LTM depends on neural activity triggered by novel or rewarding aspects of the stimulus to be encoded [126]. Our results fit well with current theories and show that the role of novelty is broader than previously considered. In accordance with our findings, Gruber et al. [127] observed that people found it easier to learn about topics that interested them. Perhaps pursuing the same basic inquiry of us, in this work, it was used fMRI to investigate how curiosity influenced memory. Healthy individuals showed improved memory formation for information that they were curious about and for incidental material learned during states of high curiosity, evaluated in both immediate and one-day-delayed memory tests. It was in those states that fMRI revealed an enhanced activity in the midbrain and in the nucleus accumbens. Moreover, it was postulated that item generalization is associated with a tight coupling between activity in hippocampus and dopaminergic midbrain areas. Authors proposed that the release of dopamine in the hippocampus strengthens the encoding of both past and present features into an integrated representation. Even more, they envisioned the process as associations between synaptic tags across trials, where the dopamine release could complete these associations contributing to the acquisition of generalization [128].

Different strategies have been used in an attempt to improve LTM expression of information learned in class. One of them consists in retrieving information several times during learning. Then, in a final test session performed a week later, the group of students which performed this retrieval practice recalled substantially more word pairs than those who did not practice the words [129]. Another way to achieve better memory performance was by transferring the benefits related to training executive functions. Goldin et al. [130] found that this intervention elicits transfer to some (but not all) facets of executive function. However, the incorporation of a novel class at the right times represents a quick nonexpensive methodology (it only requires a novel session of 20 minutes) that can be easily implemented by teachers in the school in order to improve the long-term memory expression. This kind of activities support the idea that BT might be acting in the formation of human memories as well, providing an interesting strategy to boost teaching activities by using novel pedagogic tasks to improve memory for those assignments of difficult learning.

Finally, a work was recently published contributing to reveal the process of behavioral tagging in humans, showing that strong experiences associated with a weak learning improved its LTM expression [131]. Adult humans were trained using novel images from two picture categories (animals and tools). After few minutes, they were submitted to a Pavlovian fear conditioning based on pairing electric shocks with some pictures belonging to one of the categories in a counterbalanced way, while the images from the other category were unpaired. After that, a novel series of images were shown to the subjects and a surprise recognition memory test was performed immediately, 6 h and 24 h after training. The results suggested that memory for neutral objects was selectively enhanced if other objects from the same category were paired with shock. These enhancements were observed following a period of consolidation, but not in an immediate memory test or for items strongly encoded before fear conditioning.

## 10. Concluding Remarks

The coincidence between the main attributes of memory and synaptic models of long-term plasticity, in relation to specificity and persistence, leads to the formulation of the synaptic plasticity and memory hypothesis [12]. In this review, we focused on the mechanism postulated to explain input specificity either in synaptic plasticity or in memory formation. The STC hypothesis was originated in 1997 by Frey and Morris, introducing the idea that there is a temporal window shortly after LTP induction in which PRPs are targeted selectively to activated synapses in order to establish a long-lasting form of potentiation. Ten years after the proposal of this hypothesis, it was demonstrated that an analogous BT process also operates in a living animal when a LTM is being formed from a weak experience. Following these seminal discoveries [23, 32], a wide range of scientific reports fueled the idea of tagging and capture processes either in plasticity or in memory models. Along this review, we referred to the features shared by STC and BT processes and here we list the top ten analogies between them as follows.

1. A strong event helps to establish a persistent form of plasticity associated with another weak event.
2. This mechanism is dependent on the protein synthesis induced by the strong event.
3. It is also dependent on a tag set by the weak event.
4. It was described that tags have a short half-life and PRPs possess a kinetic of synthesis and degradation, both of them displaying particular distributions in the space.
5. Thus, the strong event is effective when occurring in a critical time window around the weak one.
6. This time window is biphasic, displaying one phase before and the other after the weak event.
7. The strong event induces PRPs but if it is too close to the weak event can result in lack of promotion due to the impairment in setting or maintaining the tag of the weak training/stimulus.
8. It is required that both events activate a common neuronal population.
9. Under limited PRPs availability, a competition for capture of PRPs by different kind of tags was observed, resulting in attenuation of any of the plastic processes.
10. Similar cellular machinery and neurotransmitter systems seem to be recruited for the setting of the tag and the synthesis of PRPs in both BT and STC processes.

Consistent with these analogies, the core information presented in this review shows the effect of a novel experience on the promotion of LTM formation induced by a weak learning. This effect was explained using the BT hypothesis, which postulates that PRPs provided by novelty are used to originate LTM for a weak learning when they are captured by the specific learning tags. In the past seven years, several research groups have worked on the BT process demonstrating that it was observed in operant and Pavlovian aversive paradigms, in the formation of extinction and SOR memories as well as in other tasks based on spatial learning [32, 44, 46, 47, 53, 54, 63–65, 89, 121]. Moreover, a similar phenomenon was observed in school children who had learnt about a story or drawing [69] and in adults who learnt a list of pictures [131], suggesting the generality of the process in long-lasting memory formation. However, there is no data for motor, habit, or procedural learning where an implicit memory is established after multiple similar training sessions. In those cases, the possibility exists that the learning tag could be reinstalled in each session and the PRPs provided by metaplastic processes emerging from the summation of trails or by surrounding experiences.

This review also deepened in some aspects about the nature of learning tag, in the identification of PRPs involved in the process [43, 46, 54, 63, 64, 89], and the existence of a “maintenance tag” set lately after a persistent strong learning [121]. Finally, data was summarized related to the existence of competition for PRPs leading to memory interference as well as LTM improvements triggered by providing more PRPs through multiple strong events associated to a weak training [43].

From an adaptive perspective of memory, the BT process may have result successful in nature because weak experiences can gain meaning if they are accompanied by relevant events. This could assist animals to remember circumstances associated with experiences of high significance useful for predicting and controlling important events in the future. Under particular circumstances (low PRPs levels) this process helps the weak memory trace in detriment of the strong one. However, some very strong event could trigger redundant mechanisms of storage and the BT process could undergo without harming any memory traces.

## 11. Debating New Perspectives

So experiments described in this review were designed to test the BT hypothesis. The overall results satisfied the predictions of this model, gathering enough information to postulate that the formation of LTMs involves the setting of a learning tag, the synthesis of PRPs, and their further capture by those tags. However, could the process of LTM formation involve other plastic mechanisms? The obvious answer is yes. Regarding this, the “behavioral metaplasticity” term was recently incorporated indicating that memories can be primed by prior experience or stimulus [132–135]. This represents a behavioral adaptation from the original concept of metaplasticity defined as a prior activity in a network that will greatly influence the future probability of synaptic strengthening [136]. Parsons and Davis [133] trained rats with a single pairing, of a light pulse and shock, which resulted insufficient to induce short-term or long-term fear memory. Nevertheless, this pairing was successful to prime a future learning of another identical trial delivered later, allowing the formation of a long-lasting and robust fear LTM, through a metaplastic effect that required PKA signaling in the amygdala. Similarly, a delayed conditioning between a sound and a shock, which usually does not express LTM in rats, is susceptible to a metaplastic mechanism triggered by a stimulation that induces LTD immediately before training, to permit LTM formation [135]. The authors found that CA1 mGluR5 is critical for the acquisition of this associative memory that has a temporal processing component.

Taking these results into consideration, are BT and behavioral metaplasticity process mutually exclusive? To our vision the answer is no. A possible explanation contemplates that the first trial serves to lower the threshold for tagging and capture events induced by the later trial, promoting LTM formation. In fact there are some examples of metaplasticity affecting synaptic tagging processes: the induction by ryanodine receptor activation or synaptic activation of metabotropic glutamate receptors prolongs the durability of the synaptic tag extending the time window for associative interactions between stimuli [88]. Also priming stimulation through the activation of metabotropic glutamate receptors adjusts thresholds for functional plasticity through the local synthesis of PKM*ζ*. This metaplastic process operates within dendrite clusters [31]. Also BDNF might be itself a PRP and it might be able to orchestrate the plasticity threshold for a whole cluster of synapses and might therefore be involved in processes of metaplasticity [137]. At behavioral level, it was reported that sometimes the first training session has promoting effects on LTM formation for a further learning experience and sometimes has a negative effect on it [138]. In particular, novel exploration session immediately previous to a weak training did not promote the IA-LTM formation and impaired further promotion [41]. In this context, a possible explanation would be that OF exposure induces metaplastic changes in the network interfering with the setting or stabilization of IA learning tag. In contrast, promoting effects were found on LTM formation due to novel events experienced between 15 min to 2 hours apart from the weak learning. The fact that the promoting phenomenon is protein synthesis-dependent and operates in a temporal biphasic way, before and after the learning session, supports the notion that BT process could be involved in LTM formation. In sum, by extending the time frame in which events can be associated at a synaptic level and biasing synapses towards a plasticity-conducive state, synaptic tagging and metaplasticity provide potent mechanisms for enhancing memory quality in the brain [110]. Actually, it was presented an extended view that integrates neuronal allocation, synaptic tagging and capture, spine clustering, and metaplastic processes, tending to explain the exact sites where memories are stored [139].

In our opinion some hypothetical predictions of the BT hypothesis still remain to be addressed. Regarding the tag setting, it is not determined yet if different learning tasks set different kinds of learning tags. Neither if there are any differences in the quality and/or quantity of learning tags between different experiences intensity. Moreover, it will be interesting to test if metaplasticity affects the setting or the half-life of learning tags in a similar way as it was reported in synaptic plasticity model [88]. Also considering our postulation of “maintenance tagging,” could memory reactivation or retrieval induce a retagging of the activated inputs? Would this mechanism be involved in the reconsolidation of a memory trace? On the other hand, regarding the PRPs source in BT protocols, it would be interesting to characterize the type of event able to induce synthesis of proteins useful for LTM formation. There is some information in this matter [53, 63, 65]; however, it would be nice to perform a correlation between the synaptic plastic changes associated to the different types of learning used. Finally, other main questions are unresolved. Are learning tags being set and PRPs captured effectively at synaptic level? Does BT also operate in invertebrates as well? Given the remarkable degree of conservation of memory mechanisms observed across species widely separated by evolution, as well as data at synaptic level [140], this last question deserves investigation. These and many other questions will be probably answered in the near future. By now, BT hypothesis represents a wide framework to study and analyze memory processes, offering a consistent structure able to explain promotion, modulation, and interference in the formation of lasting memories.
